# Exposure to a Virtual Reality Mass-Casualty Simulation Elicits a Differential Sympathetic Response in Medical Trainees and Attending Physicians

**DOI:** 10.1017/S1049023X22002448

**Published:** 2023-02

**Authors:** Matthew A. Tovar, James A. Zebley, Mairead Higgins, Aalap Herur-Raman, Catherine H. Zwemer, Ayal Z. Pierce, Claudia Ranniger, Babak Sarani, James P. Phillips

**Affiliations:** 1. School of Medicine and Health Sciences, George Washington University, Washington, DC USA; 2. Department of Surgery, George Washington University, Washington, DC USA; 3. Department of Emergency Medicine, George Washington University, Washington, DC USA

**Keywords:** disaster medicine, mass-casualty incident, simulation, virtual reality

## Abstract

**Background:**

Previous studies have demonstrated the use of virtual reality (VR) in mass-casualty incident (MCI) simulation; however, it is uncertain if VR simulations can be a substitute for in-person disaster training. Demonstrating that VR MCI scenarios can elicit the same desired stress response achieved in live-action exercises is a first step in showing non-inferiority. The primary objective of this study was to measure changes in sympathetic nervous system (SNS) response via a decrease in heart rate variability (HRV) in subjects participating in a VR MCI scenario.

**Methods:**

An MCI simulation was filmed with a 360º camera and shown to participants on a VR headset while simultaneously recording electrocardiography (EKG) and HRV activity. Baseline HRV was measured during a calm VR scenario immediately prior to exposure to the MCI scenarios, and SNS activation was captured as a decrease in HRV compared to baseline. Cognitive stress was measured using a validated questionnaire. Wilcoxon matched pairs signed rank analysis, Welch’s t-test, and multivariate logistic regression were performed with statistical significance established at P <.05.

**Results:**

Thirty-five subjects were enrolled: eight attending physicians (two surgeons, six Emergency Medicine [EM] specialists); 13 residents (five Surgery, eight EM); and 14 medical students (six pre-clinical, eight clinical-year students). Sympathetic nervous system activation was observed in all groups during the MCI compared to baseline (P <.0001) and occurred independent of age, sex, years of experience, or prior MCI response experience. Overall, 23/35 subjects (65.7%) reported increased cognitive stress in the MCI (11/14 medical students, 9/13 residents, and 3/8 attendings). Resident and attending physicians had higher odds of discordance between SNS activation and cognitive stress compared to medical students (OR = 8.297; 95% CI, 1.408-64.60; P = .030).

**Conclusions:**

Live-actor VR MCI simulation elicited a strong sympathetic response across all groups. Thus, VR MCI training has the potential to guide acquisition of confidence in disaster response.

## Introduction

A disaster is defined by the United States Federal Emergency Management Agency (FEMA; Washington, DC USA) as the occurrence of a natural catastrophe, technological accident, or human-caused event that has resulted in severe property damage, deaths, and/or multiple injuries.^1^ Moreover, mass-casualty incidents (MCIs) are loosely defined as events where the demands placed on the available health care infrastructure acutely outweigh the resources available.^2^ The acute stress experienced by health care providers during these situations can precipitate psychological distress. The physiologic manifestations of this state include activation of the sympathetic nervous system (SNS).^3,4^ In the health care setting, psychological distress has been shown to result in elevated heart rate, hypervigilance, poor task performance, high distractibility, decreased working memory, and poor medical decision making.^5,6^ High-risk occupations have, for decades, trained under artificial conditions that elicit similar stress responses in order to learn to compensate for these deleterious physiologic changes and complete the required tasks. Examples include firefighters, military combatants, and law enforcement officers. Similarly, training health care professionals to excel during a mass-casualty response should include building mental resilience for receiving large numbers of critical patients, recognizing and compensating for acute physiological stress, and increasing incident management and decision-making confidence.^7^

The gold standard of MCI training is the live-action, full-scale drill. When properly engineered, these simulations have the potential to recreate the chaotic realism of a real-life MCI. However, numerous studies have suggested that in-person drills have low reproducibility, high expenses, and are limited in their effectiveness.^8–10^ As an alternative, tabletop exercises have been used as a substitute. Tabletop simulations are non-threatening discussions designed to test emergency management professionals in assigned roles without physical involvement or acute time constraints. They can be performed at a relatively low cost and have been shown to increase short-term medical provider confidence in disaster management techniques. Unfortunately, this method has not been shown to improve participant knowledge acquisition via the exercise, nor accurately recreate the MCI’s intrinsic entropic volatility.^11^ Because of these and other limitations, mass-casualty exercises are rarely given financial priority by hospitals and are often relegated to the most-minimal complexity required to complete an accreditation requirement. Ultimately, many health care facilities remain unprepared for disaster situations.^12–14^

Virtual reality (VR) technology is an area of emerging interest in critical situation training and its use has already been described in a variety of spaces such as perioperative surgical planning,^15,16^ medical and surgical education,^17,18^ nursing education,^19^ neurorehabilitation,^20^ disaster planning,^21^ and military readiness.^22^ Additionally, several studies have suggested that VR training is more interactive and immersive than conventional training.^23,24^ Therefore, VR has the potential to enhance learning and retention of disaster response and management skills and the mental rehearsal and encoding of the disaster experience.

**Figure 1.**
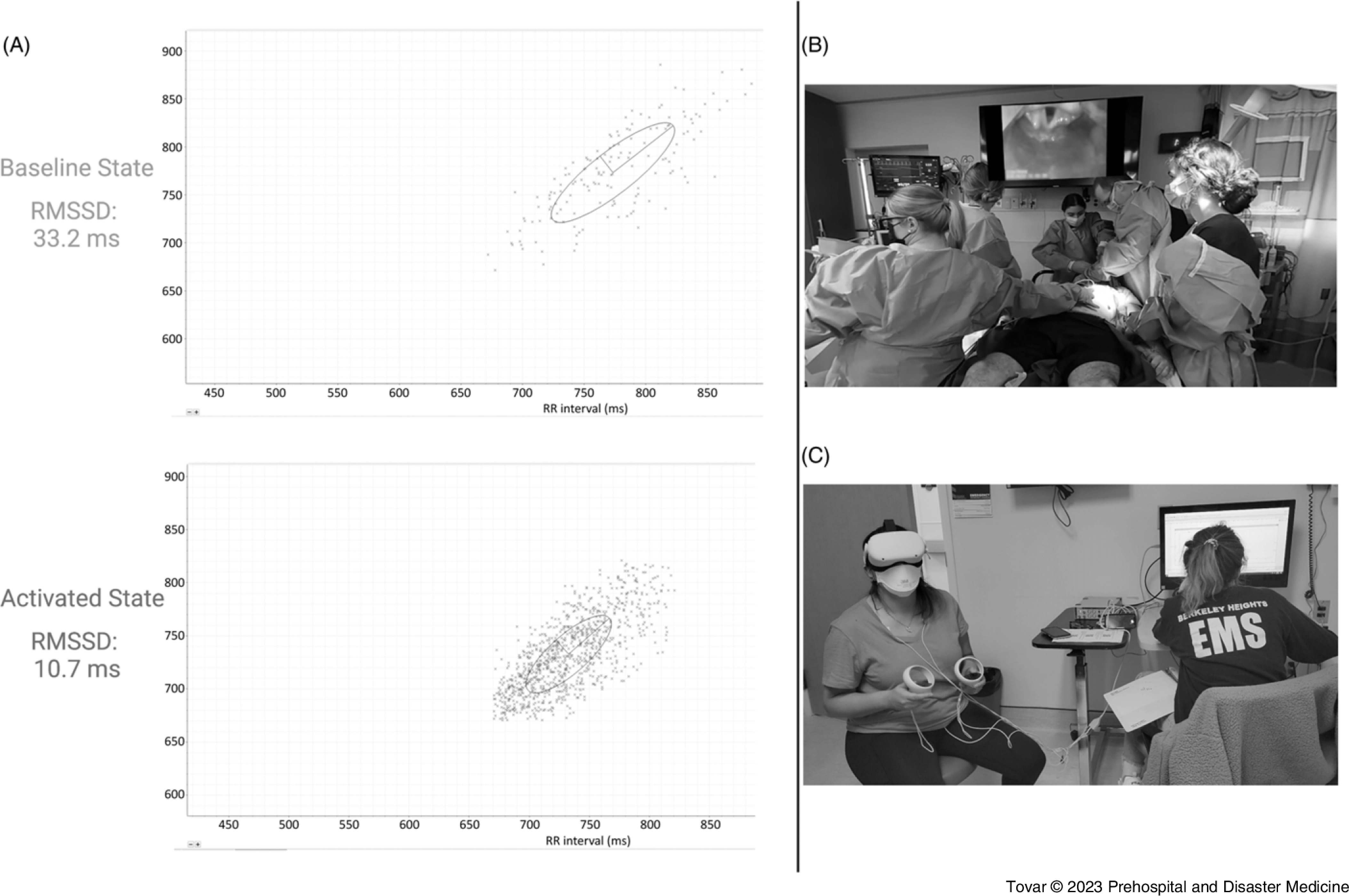
**(A):** Pair of Poincaré Plots of the R-Rn+1 Distance (in milliseconds) Plotted as a Function of the R-Rn Distance (in milliseconds) of a Subject at a Baseline Resting State (top) and an Activated State (bottom) Showcasing the Decrease in HRV as a Function of SNS Activation. **(B):** Screenshot of the Live-Actor VR MCI Simulation Showing a Physician Intubating a Mass-Casualty Patient with an Unstable Airway. **(C):** Photograph of a Participant with the VR Headset Donned and Attached to the HRV Recording Instrument via EKG Leads. Abbreviations: SNS, sympathetic nervous system; VR, virtual reality; MCI, mass-casualty incident; HRV, heart rate variability; EKG, electrocardiography; RMSSD, root-mean-squared of successive differences.

Medical providers exposed to stress exhibit characteristic physiologic and psychologic changes that can be measured. These include increase in stress-related hormone levels as well as decreases in heart rate variability (HRV).^6^ Briefly, HRV is defined as the beat-to-beat variation (in milliseconds, Msec) in cardiac electrical activity. When represented in its time-gated form, HRV can serve as a mathematical representation of the frequency of respiratory sinus arrythmia that is largely driven by parasympathetic input into the sinoatrial node.^25,26^ In general, it is surmised that a decrease in the time-gated HRV value represents relative SNS activation (Figure 1A) and an increase in the time-gated HRV value represents relative parasympathetic activation, though various exceptions to this rule exist (ie, accentuated antagonism and parasympathetic rebound).^26^ Assessing HRV, based on real-time electrocardiography (EKG), is among the most promising methods to measure stress due to its high real-time precision and non-invasive nature.^27^ These HRV measurements can provide insight into the neurophysiological stress response of a participant, but it is also necessary to evaluate psychological stress as a complementary measurement to best understand the overall human stress response. Psychological stress can be measured through validated questionnaires, including the State Trait Anxiety Inventory (STAI), a six-question, four-unit Likert-style survey.

While some areas of trauma resuscitation have incorporated VR experiences into training,^23,24^ the evaluation of this emerging technology in conjunction with measuring changes in physiological and psychological markers in the setting of an MCI is lacking. Thus, the aim of this study was to evaluate the changes in psychological and physiological stress of medical students and physicians at different stages of training, as measured by HRV and the STAI questionnaire, when immersed in a live-actor VR MCI. It was hypothesized that the immersive VR MCI environment would be able to elicit a measurable physiological response and the magnitude of that response will be correlated with the subject’s level of training. Thus, VR may be useful in measuring physiologic and cognitive confidence in those who may be called to respond to or receive casualties from a disaster scenario.

## Materials and Methods

### Study Design and Setting

This was a single-center, observational, quantitative epidemiological study performed among a pool of medical students, Emergency Medicine (EM) and Surgery resident physicians, and attending physicians at an academic, urban Level I trauma center in Washington DC, USA. This hospital trains approximately 41 EM residents and 27 Surgery residents per year, in addition to teaching approximately 600 medical students. It is staffed by a cohort of five board certified trauma surgeons and approximately 50 core EM academic physician staff and sees approximately 1,600 trauma activations per year. The protocol used in this study was approved by the University Institutional Review Board (IRB) (#NCR213858). All individuals gave informed written consent approved by the IRB to participate in this study prior to enrollment.

### Selection of Participants

To determine the proper sample size to detect a 10%-unit difference via Wilcoxon’s matched pairs test in the primary outcome (HRV) with 1-β = 0.8 and α = 0.95, an *a-priori* power analysis was performed using G*Power computer software (version 3.1.9.6; Institut für Psychologie, Christian-Albrechts-Universität Kiel; Kiel, Germany). The results returned a minimum sample size of 35 subjects (β_actual_ = 0.8070) required to properly power this study.

Subjects were invited to participate in this study via email and in-person recruitment. All subjects were asked to take an inclusion/exclusion survey (Supplemental Material 1 for survey contents; available online only) prior to formal enrollment, which took place from February 2022 through May 2022. Inclusion criteria were an age of 18 years or older and currently enrolled either in medical school, Accreditation Council for Graduate Medical Education (ACGME; Chicago, Illinois USA)-approved residency program for General Surgery or EM, or a licensed attending physician working in the Departments of Surgery or EM. Exclusion criteria were a known history of severe vertigo or motion sickness, neuro-vestibular or neurovascular disease, epileptiform disease, posttraumatic stress disorder, severe migraines, claustrophobia, hemophobia, and failure to complete a motion-sickness trial (Epic Rollercoaster; B4T Games; Brazil). After enrollment, various demographic information was collected (Supplemental Material 2 for demographic survey contents; available online only) and included information regarding the stage of medical education, age, gender, past military or first responder history, and past real-life MCI response experience.

### Creating the Simulated MCI

A total of two trauma scenarios were created, both were inspired by standardized cases described in trauma resuscitation training courses. A GoPro 360 camera (Version 1, GoPro; San Mateo, California USA) was mounted on the shoulder of a “trauma lead” physician, which was played by a board-certified trauma surgeon (author BS) standing at the foot of the bed (Figure 1B). The trauma team also included a senior General Surgery resident (author JAZ) performing the primary and secondary survey, a medical student with former trauma nurse training and experience, and Emergency Medical Technicians cross-trained in in-hospital trauma resuscitation. All personnel underwent a four-hour orientation prior to filming the resuscitation. The simulation was conducted in the high-fidelity simulation lab of the medical school.

In post-production, footage was uploaded to a Razer Blade 15 Advanced laptop (8th Gen Intel Core i7-8750H CPU @ 2.20GHz, 16.0GB RAM, NVIDIA GeForce RTX 2080 Max-Q GPU; Razer; Irvine, California USA) running Windows 10 Home (Version 21H2, Microsoft; Redmond, Washington USA). Additional auditory stimuli were prepared via Adobe Audition (Version 23.0, Adobe; San Jose, California USA) and included bedside alarms, emergency vehicle sirens, and helicopters blades. These stimuli were added to the videos via Adobe Premier (Version 23.0, Adobe; San Jose, California USA). Then, footage was converted to 4K equirectangular MP4 360° video format using the H.264 codec via the free GoPro Player software along with the HEVC Video Extensions (Version 2.0.52911; Microsoft; Redmond, Washington USA) plugin. Unity (Version 2022.1.23, Unity Technologies; San Francisco, California USA) 3D software was utilized to create a VR scene to view the processed 360° video. A virtual 3D sphere was created in Unity and a custom shader was used to project the 360° video onto the inside of the sphere. Using the Meta XR-Plugin for Unity, the scene was viewed in VR on the Meta Quest 2 (Version 45.0, Meta; Menlo Park, California USA) VR head mounted display (HMD).

### Measurements

After enrollment and appropriate informed consent was obtained, research subjects were oriented to the Meta Quest 2 headset. Non-invasive adhesive electrocardiogram electrodes were attached to the subject using a modified Mason-Likert lead placement to minimize potential background noise. Subjects’ baseline heart rate and HRV were attained while sitting in a quiet, non-stimulating environment for two minutes. After baseline data collection, subjects were exposed to the following four VR conditions (Figure 1C):

1. VR Non-Stressful Non-Medical Control: “*Nature Treks VR”* was an interactive walk-through simulator in which participants can visit and walk through various virtual environments. Subjects were immersed in the “*Green Meadow”* simulation for two minutes.
2. VR Stressful Non-Medical Control: *“Mission ISS”* was an interactive VR tour of the International Space Station (ISS). The subject was placed in a spacewalk and had to orient and maneuver in outer space using a Manned Maneuvering Unit without straying too far from the station. Subjects were immersed in this environment for two minutes.
3. VR Trauma Resuscitation – Low-Grade Trauma: This simulation involved evaluating and treating an intoxicated, confused driver that struck a tree and sustained non-life-threatening injuries. The patient appeared hemodynamically stable in the primary and secondary survey and had a negative focused assessment for the survey of trauma (FAST) exam.
4. VR Trauma Resuscitation – High-Stress: This simulation involved the subject evaluating and treating four back-to-back high-grade traumas from a simulated MCI shooting (Figure 1B). Injuries included a gunshot wound to the neck with an unstable airway, traumatic pneumothorax, traumatic hemothorax, and visceral organ injury resulting in intra-abdominal bleeding. This scenario also involved an elevated degree of auditory stimuli including sustained background chatter representing an overwhelmed medical unit taking trauma casualties, sustained alarms of medical monitors, and the cries for help of patients being treated. Visual stimuli included frank blood loss, acting as team leader in a room filled with medical personnel, and the visual cues of numerous severely injured patients.

The scenarios were shown in sequence from one (1) to four (4) to minimize the bias of residual SNS activation from prior scenarios. The subject’s heart rate and HRV were continuously recorded throughout all four evolutions utilizing LabChart (Version 8.1.24, ADInstruments; Sydney, Australia). Immediately after each evolution, subjects were asked to complete the STAI survey (Supplemental Material 3 for STAI survey contents; available online only).^28,29^ Discordance between SNS activation and cognitive stress was defined as having significant SNS activation but no reported changes in cognitive stress by the STAI survey. Heart rate variability was calculated by measuring the R-R interval on lead II of the EKG tracing. To attain this value, the root-mean-squared of successive differences (RMSSD) was obtained as described by Shaffer, et al.^26^

### Analysis

The RMSSD value of each subject was divided over the subject’s mean baseline RMSSD value, creating a fold-change representation of their RMSSD (Δ-RMSSD). Given that no validated definition exists for the magnitude of Δ-RMSSD required to qualify as significant SNS activation, internal data collection was performed that included measuring baseline and control HRV values among research personnel to approximate appropriate cutoff values. Data preparatory to research revealed that HRV decreased no more than five percent relative to a person’s baseline HRV when exposed to the controls used in this study. Thus, a “significant” change in HRV was defined as four-times the expected change in HRV observed in the internal data collection, or a 20% change relative to a subject’s baseline state. Data were analyzed using the Wilcoxon matched-pairs test, Welch’s t-test, Fischer’s exact test, one way ANOVA, and Tukey’s ANOVA test as appropriate. A multivariate logistic regression model was established by dichotomizing SNS activation along the 20% cutoff relative to baseline and analyzing the effect that each metric detailed in Supplemental Material 2 had on the odds of significant SNS activation. Statistical significance was defined as P <.05. All data were analyzed utilizing Prism (v09.3.0, GraphPad Software; San Diego, California USA).

## Results

### Subject Demographics

A total of 27 surgical residents, 41 EM residents, 50 EM faculty, eight trauma surgery faculty, and 600 medical students were invited to participate. Overall, 35 participants responded and were enrolled into the study. These included 14 (2.1%) medical students, five (18.5%) Surgery residents, eight (26.8%) EM residents, two (25%) trauma surgeons, and six (12%) EM faculty. Of the 14 medical students, four were first-year students, two were second-year students, six were third-year students, and two were fourth-year students. Of the five General Surgery residents, one was a second-year resident, two were third-year residents, and two were fourth-year residents. Of the eight EM residents, four were first-year residents, one was a second-year resident, one was a third-year resident, and two were fourth-year residents. No subjects were recorded with an incomplete data set.

The demographics of each study group are shown in Table 1. The average medical student, resident, and attending ages were 27.4 (SD = 4.35) years, 29.0 (SD = 1.87) years, and 42.4 (SD = 7.84) years, respectively. Compared to attending physicians, both medical students and residents were significantly younger (P <.0001). The gender distribution was approximately equal across all groups, with 72% of participating medical students identifying as female, 62% of residents identifying as female, and 63% of attendings identifying as female (P = .694). The average duration of trauma-related experience among medical students, residents, and trainees were 3.15 (SD = 3.1) years, 4.92 (SD = 1.8) years, and 16.2 (SD = 6.52) years, respectively. Compared to attending physicians, both medical students and resident physicians had significantly less medical experience (P <.0001).

**Table 1.** Demographic Data of Participants

	MS n = 14	RES n = 13	ATT n = 8	P Value
Age, Years (SD)	27.4 (SD = 4.35)	29.0 (SD = 1.87)	42.4 (SD = 7.84)	P <.0001^*^
Gender: n (%)	M: 4/14 (28%) F: 10/14 (72%)	M: 5/13 (38%) F: 8/13 (62%)	M: 3/8 (37%) F: 7/8 (63%)	Post-Hoc^∇^
Trauma Experience, Years (SD)	3.15 (SD = 3.10)	4.92 (SD = 1.80)	16.2 (SD = 6.52)	P <.0001^*^
Deployed/FR History (%)	4/14 (28%)	3/13 (23%)	5/8 (62%)	Post-Hoc ^∇^
History of Prior MCI Response (%)	1/14 (7.1%)	1/13 (7.6%)	4/8 (50%)	**Post-Hoc^∇^**

Post-Hoc Analysis:			
	MS vs. RES	MS vs. ATT	RES vs. ATT
Age^§^	P = .652	**P <.0001**	**P <.0001**
Gender^∇^	P = .694	P >.999	P >.999
Trauma Experience^§^	P = .450	**P <.0001**	**P <.0001**
Deployed/FR History^∇^	P >.999	P = .3864	P = .3458
History of Prior MCI Response^∇^	P >.999	**P = .0393**	**P = .0475**

Abbreviations: MCI, mass-casualty incident; MS, medical students; RES, residents; ATT, attendings; FR, first responder.

* = Ordinary One Way ANOVA.

∇ = Fishers Exact test.

§ = Tukey’s multiple comparison test.

Deployment and first responder history was also similar among the three cohorts, with 31% of medical students reporting deployment/first responder experience compared to 20% of residents and 50% of attending physicians (P = .187). Finally, only one medical student reported having prior MCI response experience compared to one resident physician and four attending physicians. This difference was significant between medical students versus attendings (P = .039) and residents versus attendings (P = .047). No significant differences were observed between EM physicians and surgeons (data not shown) across any demographic factor.

### MCI Immersion Resulted in Increased Sympathetic Tone Relative to Baseline and the Magnitude of Activation was Correlated to Trainee Level

**Figure 2.**
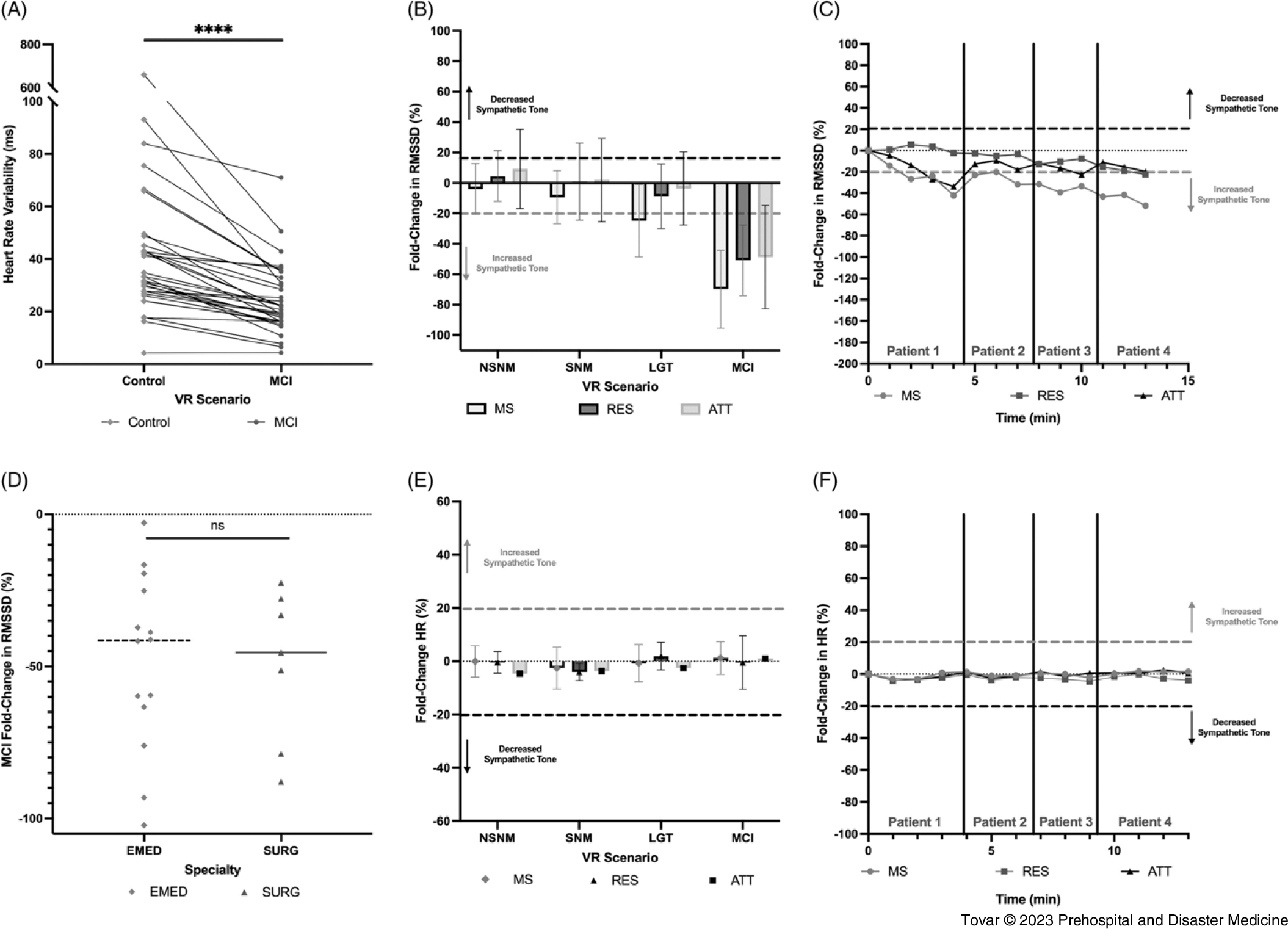
**(A):** Paired Analysis Graph Showcasing the Significant Drop in HRV when Viewing the MCI Relative to the Baseline Control. **(B):** Grouped Bar Graph Showing the Magnitude of Sympathetic Activation is Greatest in MS, followed by RES and ATT. MS also had Significant Sympathetic Activation when Viewing the LGT while RES and ATT Did Not. **(C):** Plot of Fold-Change RMSSD (Δ-RMSSD) Plotted as a Function of Time in the MCI Showing that MS Stayed Persistently Activated while RES and ATT Regressed to their Baseline State. **(D):** Dot Plot Comparing the Δ-RMSSD of EMED Physicians to SURG Showing No Significant Difference in the Magnitude of Sympathetic Activation between the Two Specialties. **(E):** Grouped Bar Graph Showing No Significant Change in HR of Any Group while in Any Control Condition. **(F):** Plot of the Change in HR versus Time Showing there was No Significant Change in HR in Any Group as they Progressed through the MCI. Abbreviations: HRV, heart rate variability; VR, virtual reality; MCI, mass-casualty incident; RMSSD, root-mean-squared of successive differences; MS, medical students; RES, residents; ATT, attendings; LGT, low-grade trauma; NSNM, non-stressful non-medical; SNM, stressful non-medical; HR, heart rate; EMED, Emergency Medicine; SURG, surgeons.

There were no significant differences in the baseline RMSSD values between medical students (80.5 [SD = 67] Msec), residents (34.7 [SD = 19.6] Msec), and attendings (44.7 [SD = 28.1] Msec); P = .4420). Overall, 34/35 subjects (97.1%) experienced a statistically significant decrease in HRV (P <.0001) in the MCI simulation, including 14/14 medical students (100%; P = .0002), 13/13 resident physicians (100%; P = .0001), and 7/8 attending physicians (87.5%; P = .015); Figure 2A. These findings indicated increased SNS activation when viewing the MCI compared to the subject’s baseline state. The mean Δ-RMSSD was below the 20% threshold for SNS activation for all three subgroups, with medical students exhibiting the greatest change from baseline (Δ-RMSSD = −69.8%), followed by residents (Δ-RMSSD = −59.8%) and attending physicians (Δ-RMSSD = −48.7%).

Overall, 12/35 subjects (34.3%) experienced a statistically significant (P = .002) decrease in HRV when viewing the low-grade trauma, including 6/14 (42.8%) medical students, 4/13 (30.7%) resident physicians, and 2/8 (25.0%) attending physicians. Comparison of the Δ-RMSSD values averaged over each of the three cohorts indicated that, overall, the medical student cohort exhibited the significant SNS activation (Δ-RMSSD = −24.8%) whereas the resident and attending physician cohorts did not (Δ-RMSSD = −8.74%, −3.64%, respectively). No cohort exhibited significant SNS activation in the non-stressful non-medical control or the stressful non-medical control (Figure 2B).

Analysis of Δ-RMSSD over time revealed bimodal maximal activation periods, with medical students and attending physicians experiencing a peak of maximal activation at minute four of the MCI, and all three groups experiencing a second peak of maximal activation at minute 13. These correspond to caring for patient one (minute four; a gunshot wound victim with an unstable airway) and patient four (minute 13; a gunshot wound victim with intra-abdominal bleeding and aggression); Figure 2C. Medical students stayed persistently activated throughout the entire MCI evolution, not deviating above Δ-RMSSD = −20% cutoff past minute two of the MCI, whereas resident and attending physicians regressed to their baseline state following minute four of the MCI and stayed within their baseline range until minute 13 (Figure 2C). Comparison of EM physicians and surgeons revealed no significant difference in the Δ-RMSSD values between the two subgroups when viewing the MCI simulation (-48.3% [SD = 29.9%] versus −49.5% [SD = 25.2%], respectively; P = .57; Figure 2D). While there was a significant change in RMSSD values in the MCI evolution among all three groups of subjects, there were no significant changes in heart rate in any of the control conditions or at any point in the MCI condition (Figure 2E, Figure 2F). These findings indicate that the observed decrease in RMSSD occurred independent of the subjects’ heart rate.

### Multivariate Logistic Regression for Significant SNS Activation

A multivariate logistic regression model was created for both the MCI and the low-grade trauma scenario to determine what demographic factors are associated with increased odds of SNS activation. The model failed to yield any results for the MCI simulation as only one subject did *not* undergo significant SNS activation. The logistic regression model for the low-grade trauma showed that age, gender, trauma experience, completion of medical school, history of deployment or first responder experience, or history or prior MCI experience could *not* be utilized to predict increased odds of SNS activation, as shown in Table 2.

**Table 2.** Adjusted and Unadjusted Odds Ratios (OR) for SNS Activation in All Participants

Metric	Unadjusted OR (95% CI)	Adjusted OR (95% CI)	Adjusted OR P Value
Age	0.738 (0.296-0.796)	0.231 (0.002-24.610	.552
Male Gender	1.531 (0.344-6.15)	1.660 (0.330-8.644)	.534
Trauma Experience	0.840 (0.370-0.906)	0.996 (0.783-1.256)	.974
Completion of Medical School	0.467 (0.112-1.826)	0.325 (0.047-1.898)	.222
Deployed/FR History	0.481 (0.117-2.018)	0.388 (0.051-2.158)	.306
History of Prior MCI Response	0.900 (0.151-4.781)	1.096(0.0621-14.020)	.944

Abbreviation: SNS, sympathetic nervous system; FR, First Responder; MCI, mass-casualty incident.

### Discordance Between Objective SNS Activation and Reported Acknowledgement of Increased Psychological Stress

A total of 23/35 subjects (65.7%) reported increased cognitive stress in the MCI, including 11/14 (78.5%) medical students, 9/13 (69.2%) resident physicians, and 3/8 (37.5%) attending physicians. Attending physicians exhibited the highest rate of discordance between SNS activation and cognitive stress (3/8, 37.5%), followed by resident physicians (3/13, 23%) and medical students (2/14, 14.2%). A logistic regression model (Table 3) of demographic factors associated with increased odds of discordance between physiological SNS activation and cognitive acknowledgement of stress also suggested that those having completed medical school had greater odds of exhibiting physiological/cognitive discordance (OR = 8.297; 95% CI, 1.41-64.60; P = .030), given all other factors held constant. Similar findings were also appreciated in comparing the incidence of physiological/cognitive discordance in the low-grade trauma (OR = 48.16; 95% CI, 4.23-1.87x10^3^; P = .0083). Multicollinearity investigation and *post-hoc* sensitivity analysis (One Factor at a Time, OFAT; Supplemental Material 4; available online only) revealed that sequential manipulation of each variable slightly modified the overall effect size in both the MCI and the low-grade trauma scenarios; however, the overall argument that those having completed medical school had increased odds of physiological/cognitive discordance remained valid across all sensitivity analyses. Additionally, multicollinearity analysis suggested that each covariate in this model was suitable and not codependent for analysis by multivariate logistic regression. Age, gender, trauma experience, deployment history, or prior MCI response could not be used to predict increased odds of physiological/cognitive discordance.

**Table 3.** Adjusted and Unadjusted Odds Ratios (OR) for SNS/Cognitive Discordance in All Participants

Metric	Unadjusted OR (95% CI)	Adjusted OR (95% CI)	Adjusted OR P Value
Age	0.974 (0.881-1.066)	0.956 (0.782-1.153)	.631
Male Gender	0.694 (0.154-2.757)	1.867 (0.339-12.030)	.480
Trauma Experience	0.972 (0.865-1.079)	0.919 (0.719-1.144)	.458
Completion of Medical School	**3.375 (0.805-16.23)**	**8.297 (1.408-64.60)**	**.030**
Deployed/FR History (%)	1.100 (0.262-4.641)	1.390 (0.719-1.144)	.701
History of Prior MCI Response (%)	1.731 (0.249-14.72)	1.741 (0.153-21.94)	.649

Abbreviation: SNS, sympathetic nervous system; FR, first responder; MCI, mass-casualty incident.

## Discussion

Full-scale disaster exercises are a requirement for hospital accreditation, but almost all disaster exercise efforts lack the resources and stakeholder support necessary to create realistic, emotion-provoking scenarios that test skills under pressure and identify gaps in training and resource procurement. Live-action exercises are expensive, disrupt health care operations, and require significant personnel resources including trained actors, observers, moulage experts, and health care workers that often participate while off shift and unpaid. As a result, these exercises are typically poorly supported by health care administration and staff alike. This proof-of-concept study is the earliest phase of a long-term aim to demonstrate that VR MCI training can be validated as a standardizable adjunct, or even substitute, for live-action disaster training exercises. The results from this study showed that VR MCI simulation induced SNS activation across all three subject groups and that the degree of change HRV correlated with the participant’s level of training. These findings suggest that there is a measurable physiologic response in the VR simulation, which is a first step in demonstrating simulation non-inferiority between VR MCI training and live-action exercises.

Emotionally arousing and stressful experiences are known to enhance memory formation through a complex series of neural pathways;^30–32^ HRV is a validated and measurable means of assessing physiologic stress.^31–36^ As the overarching goal of medical simulation is to encode memories that providers can then recall during actual patient experiences, by activation of the SNS and recruitment of the limbic system, VR-delivered simulation has the potential to enhance the encoding and consolidation of disaster and mass-casualty training. Prior studies have suggested that HRV-guided stress inoculation training resulted in less SNS activation compared to a control group when exposed to an arousing combat scenario in a group of military servicemembers.^31,37^ It is postulated that the same effects might be seen in cohorts of front-line medical personnel should a similar HRV-guided inoculation training platform focused on disaster response be created.

The poor correlation between physiological and cognitive markers of stress in physicians, particularly trauma surgeons, has been previously described by Joseph, et al.^28^ This finding is again shown in this study with both surgeons and EM specialists exhibiting poor correlations between physiological and cognitive markers of stress. These findings support the original hypothesis by Joseph, et al that trauma resuscitation has significant physiologic mental strain that is attenuated by a physician’s sense of confidence and control.^28^ The authors further add that this sense of confidence and control in the chaos of trauma resuscitation develops sometime in the residency training process, as medical students did not express discordance between physiological and cognitive stress markers, but resident physicians did. Despite the authors’ efforts at employing best practices to control for confounding bias (Supplemental Material 4),^38,39^ it is acknowledged that potential unrecognized and thus uncontrolled confounders exist that could limit the generalizability of these findings. Furthermore, it is recognized that the 95% confidence interval of the odds ratio is very wide, likely stemming from a small number of outcome events. Thus, this association should be interpreted with statistical caution, and larger studies would be required to generate a more precise effect estimate. Nonetheless, by adding realistic and immersive visual and auditory stimuli to the process of rehearsing for MCIs, the authors purport that VR simulation can assist in helping medical students and resident physicians attain higher levels of confidence in leading MCI resuscitations.

## Limitations

It has previously been shown that HRV, specifically representations of HRV that report Fourier-transformed frequency-domain metrics, are subject to confounding from exercise, postural changes, and certain respiratory patterns.^40–44^ To reduce these unwanted effects, time-domain HRV measurements (RMSSD), which have been shown to be less affected by respiratory patterns than frequency-domain metrics,^26,45,46^ were used in this study. Other limitations include that all subjects were derived from a single institution. It is unknown whether trainees and physicians from areas with other injury patterns would have exhibited different sympathetic responses to the scenarios shown. In addition, the scenarios included coworkers of the recruited subjects. This could have led to either an exaggerated sympathetic response if there were close personal relations between the subject and a mock patient or an attenuated sympathetic response as many physicians anecdotally described feelings of calm attentiveness between themselves and the trauma team lead. Despite these limitations, this study objectively showed that live actor VR-based MCI training has the capacity to activate the SNS in all subjects with implications to improve disaster training in a reproducible and distributable manner.

## Conclusion

Preparation and training for MCIs remains difficult. This study demonstrated the ability of a live-actor VR MCI training platform to induce SNS activation at all levels of training, from medical student to attending physician. Further research is needed to validate live-actor VR-based training scenarios as a means of assessing MCI response readiness and to establish training noninferiority compared to live-action MCI exercises.

## Supplementary material

For supplementary material accompanying this paper visit https://doi.org/10.1017/S1049023X22002448. click here to view supplementary material
