# Supplementary material for: Exposure to a Virtual Reality Mass-Casualty Simulation Elicits a Differential Sympathetic Response in Medical Trainees and Attending Physicians
Source: Prehosp Disaster Med. 2023 Jan 6;38(1):48–56. doi: 10.1017/S1049023X22002448 (PMC9885434; doi:10.1017/S1049023X22002448)
Supplement: Supplementary file 1 [file S1049023X22002448sup001.docx]

**Exposure to a Virtual Reality Mass Casualty Simulation Elicits a Differential Sympathetic Response in Medical Trainees and Attending Physicians**

Matthew A. Tovar, James A Zebley MD, Mairead Higgins, Aalap Herur-Raman MS, Catherine H. Zwemer, Ayal Z. Pierce MD, Claudia Ranniger MD PhD, Babak Sarani MD, James P. Phillips MD

Electronic Supplemental Information

**Supplemental Section 1: Contents of Inclusion/Exclusion Survey**


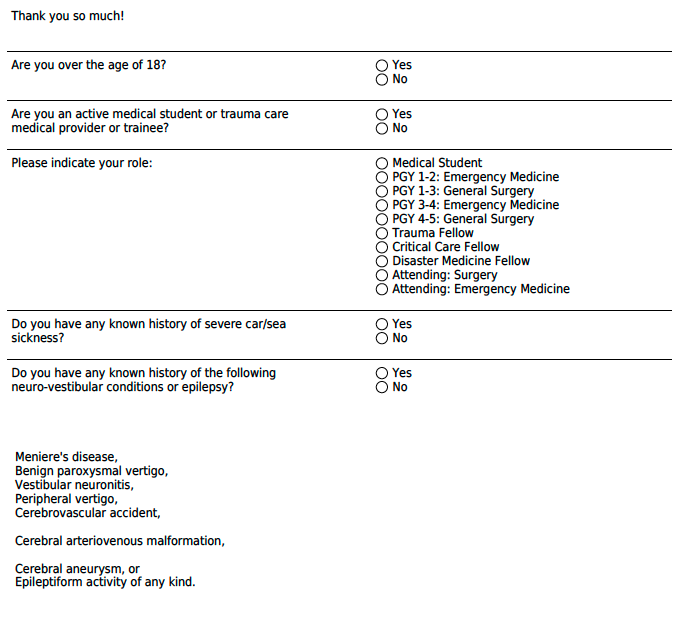


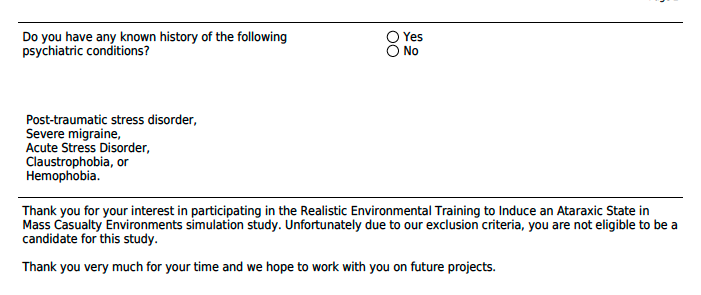


**Supplemental Section 2: Contents of Demographic Survey**


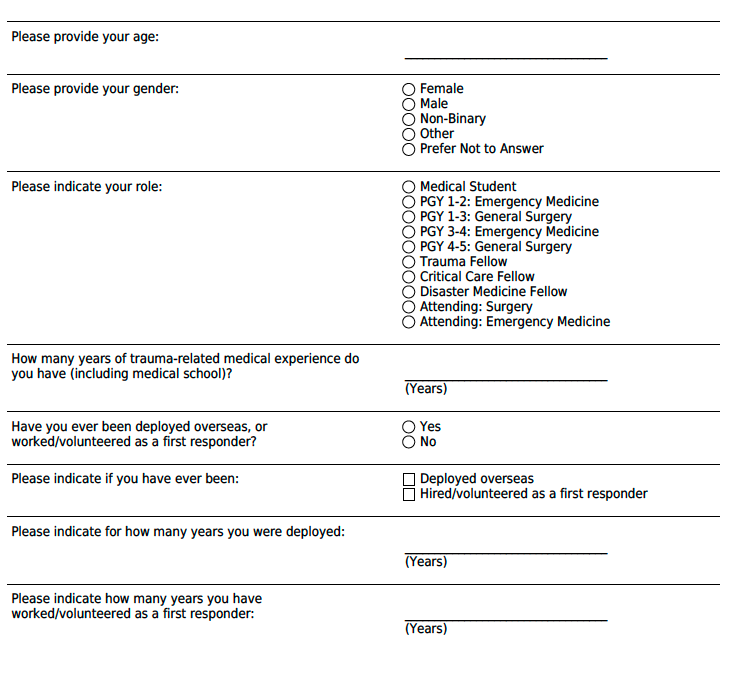


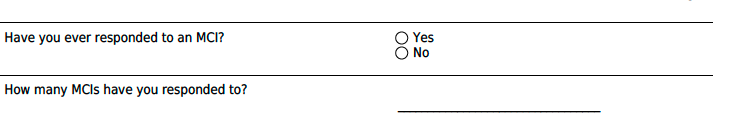


**Supplemental Section 3: Contents of State Trait Survey Anxiety Inventory**

**
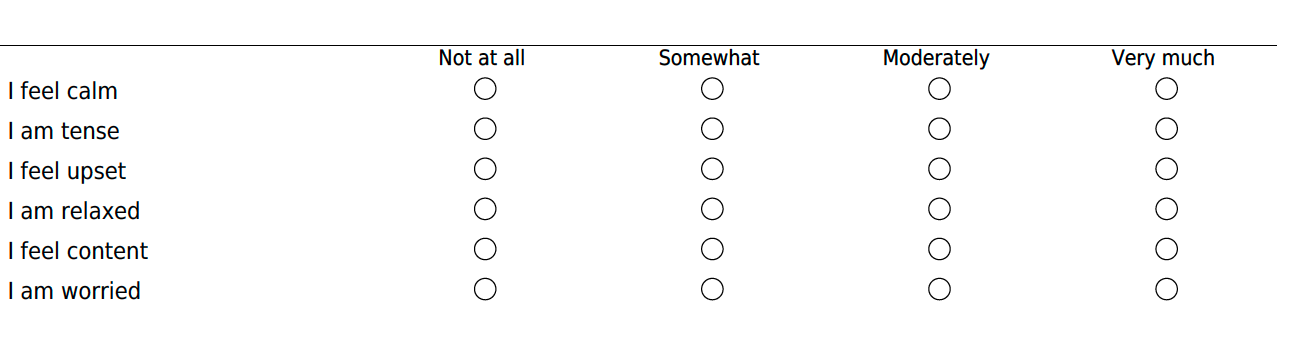
**

**Supplemental Section 4: Suitability and Validation of the Physiological/Cognitive Multivariate Logistic Regression Model**

Suitability and validation of the multivariate logistic regression (MLR) model presented in Table 3 was tested *post hoc* using two different methods: multicollinearity was calculated and reported to show appropriate suitability of each predictor variable in the overall model, and One Factor At a Time (OFAT) analysis was utilized to determine the effects of each variable on the overall statistical outcome. These *post hoc* analyses serve to fortify the logistic regression model and each of its constituent predictor variables.

*Multicollinearity*

Multicollinearity (i.e., the phenomenon in which one predictor variable is linearly related to others) was estimated first by calculating the coefficient of determination ($R_{j}^{2}$) of a regression of predictor value, *j*, on all other predictor values. The inverse of 1-$R_{j}^{2}$, known as the variance inflation factor (VIF; equation 1) quantified the severity of multicollinearity for each predictor value.

| $VIF= \frac{1}{1-R_{j}^{2}}$ | (1) |
| --- | --- |

where VIF is the variance inflation factor and $R_{j}^{2}$ is the coefficient of determination. A threshold of VIF >10 has previously been described as having considerable collinearity; however, other studies have suggested a VIF value of 5 as a more conservative threshold estimate.^1–4^ The calculated $R_{j}^{2}$ and VIF values of the predictor values in this study (shown in table S1) indicate that multicollinearity was below the thresholds of 5 and 10 in all cases, suggesting that each factor was suitable and not codependent for the MLR model.

| **Supplementary Table S1** | | |
| --- | --- | --- |
| Predictor Value | $R_{j}^{2}$-Value | VIF |
| (A) Age | 0.709 | 3.438 |
| (B) Male gender | 0.070 | 1.076 |
| (C) Trauma Experience | 0.738 | 3.817 |
| (D) Completion of Medical School | 0.280 | 1.390 |
| (E) Deployed/FR History | 0.254 | 1.342 |
| (F) Prior MCI Response | 0.200 | 1.250 |

**Supplementary Table S1:** A tabulation of the $R_{j}^{2}$-values and VIF values of each predictor value analyzed in this study showed that no moderate or significant multicollinearity was present in the MLR model.

Overall, the two predictor values most associated with each other appear to be age and years of trauma experience with VIF values of 3.438 and 3.817, respectively. This makes intuitive sense as one would attain more trauma experience as one increases in age; however, this collinearity was not significant enough to affect the overall MLR model.

*One Factor At a Time Analysis (OFAT)*

The OFAT analysis served to indicate what factors if any, when omitted from the analysis, would significantly change the statistical conclusions derived from the MLR model. The results, shown in table S2, indicate that although the effect size of the odds ratio changed slightly with iterative removal of each predictor value, there was still a statistically significant increase in the odds of physiological/psychological discordance in those who have completed medical school. This suggests that there were no “critical parameters” in the MLR model that, when removed, would invalidate the statistical argument made in this study.

| **Supplementary Table S2** | | | | | | |
| --- | --- | --- | --- | --- | --- | --- |
| **One Factor At a Time (OFAT) Analysis:** | | | | | | |
| **Metric Removed** | **A** | **B** | **C** | **D** | **E** | **F** |
|  | **aOR (95% CI)** | | | | | |
| (A) Age | 0.738  (0.296-0.796) | 0.965  (0.797-1.161) | 0.898  (0.764-1.012) | 0.958  (0.798-1.139) | 0.944  (0.757-1.147) | 0.955  (0.782-1.153) |
| (B) Male gender | 1.678  (0.323-9.77) |  | 2.144  (0.396-14.79) | 1.641  (0.353-8.340) | 1.960  (0.348-13.59) | 1.867  (0.339-12.03) |
| (C) Trauma Experience | 0.881  (0.734-1.012) | 0.915  (0.713-1.144) |  | 1.001  (0.817-1.225) | 0.928  (0.723-1.163) | 0.919  (0.719-1.144) |
| (D) Completion of Medical School | **7.851**  **(1.32-60.95)*** | **7.582**  **(1.302-56.52)*** | **6.894**  **(1.233-51.7)*** |  | **8.048**  **(1.349-63.2)*** | **8.297**  **(1.408-64.6)*** |
| (E) Deployed/FR History | 1.285  (0.214-8.372) | 1.319  (0.218-8.675) | 1.040  (0.185-5.855) | 0.965  (0.175-5.188) |  | 1.390  (0.258-8.153) |
| (F) Prior MCI Response | 1.460  (0.137-16.99) | 1.574  (0.146-18.85) | 2.027  (0.198-24.55) | 2.114  (0.239-22.80) | 1.741  (0.153-21.94) |  |

**Supplementary Table S2**: One Factor At a Time (OFAT) critical variable analysis of the multivariate logistic regression model. Columns A-G correspond to the respective variable removed (also shaded in black) from the left-most column. aOR = adjusted odds ratio of physiological/cognitive discordance adjusted for all predictor values minus the omitted value. *=p<0.05

References

1. Menard S. Applied Logistic Regression Analysis. SAGE Publications; 1995.

2. James G, Witten D, Hastie T, Tibshirani R. An Introduction to Statistical Learning: with Applications in R. Springer Science & Business Media; 2013.

3. Vittinghoff E, Glidden DV, Shiboski SC, McCulloch CE. Regression Methods in Biostatistics: Linear, Logistic, Survival, and Repeated Measures Models. Springer Science & Business Media; 2012.

4. Allison PD. Multiple Regression: A Primer. Pine Forge Press; 1999.
